# Enucleation of giant esophageal schwannoma of the upper thoracic esophagus: reports of two cases

**DOI:** 10.1186/1477-7819-12-39

**Published:** 2014-02-19

**Authors:** Hyun Woo Jeon, Kyung Soo Kim, Kwan Yong Hyun, Jae Kil Park

**Affiliations:** 1 Department of Thoracic and Cardiovascular Surgery, Bucheon St. Mary’s Hospital, The Catholic University of Korea, Bucheon, South Korea; 2 Seoul St. Mary’s Hospital, The Catholic University of Korea, Seoul Seo Cho Gu Ban Po Dong Banpo road 222, Seoul 137-701, South Korea

**Keywords:** Enucelation, Esophagus, Schwannoma

## Abstract

Benign esophageal tumors are uncommon, leiomyomas being the most frequent. However, esophageal schwannomas are exceedingly rare. We report here on two instances of large esophageal schwannomas treated by enucleation. A 63-year-old male and a 32-year-old female were referred to us for abnormal chest X-rays. Computed tomography of the chest documented sizeable growths in the upper thoracic esophagus, resulting in compression of membranous trachea posteriorly. By positron emission tomography, the tumors appeared hypermetabolic. In both instances, successful surgical enucleation was achieved. Histologic examination confirmed spindle cell tumors positive for S-100 protein by immunostaining.

## Correspondence

Benign esophageal tumors are uncommon and only 1% are detected clinically. Schwannomas are neurogenic tumors typically originating in the mediastinum. Esophageal schwannomas seldom occur and are difficult to diagnose by imaging. The sole treatment for such benign lesions is enucleation, which proved successful in the two patients reported here, each of whom presented with large esophageal schwannomas.

### Case presentation

#### Patient 1

**Figure 1:**
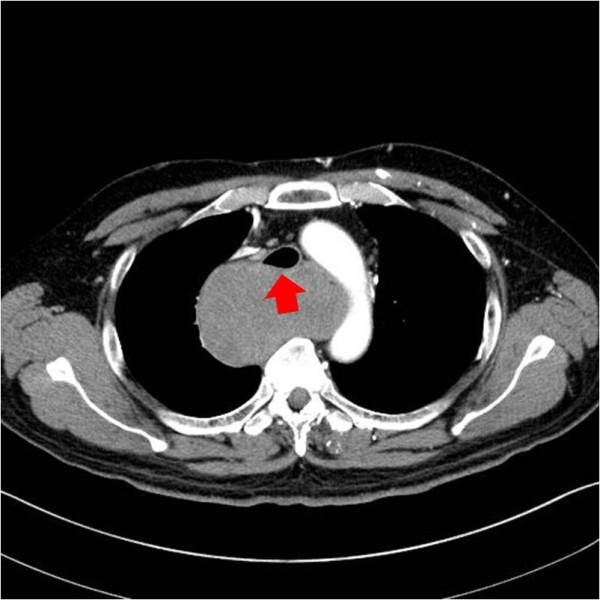
The chest CT revealed a heterogeneous large mass in the esophagus and tracheal compression (arrow).

**Figure 2:**
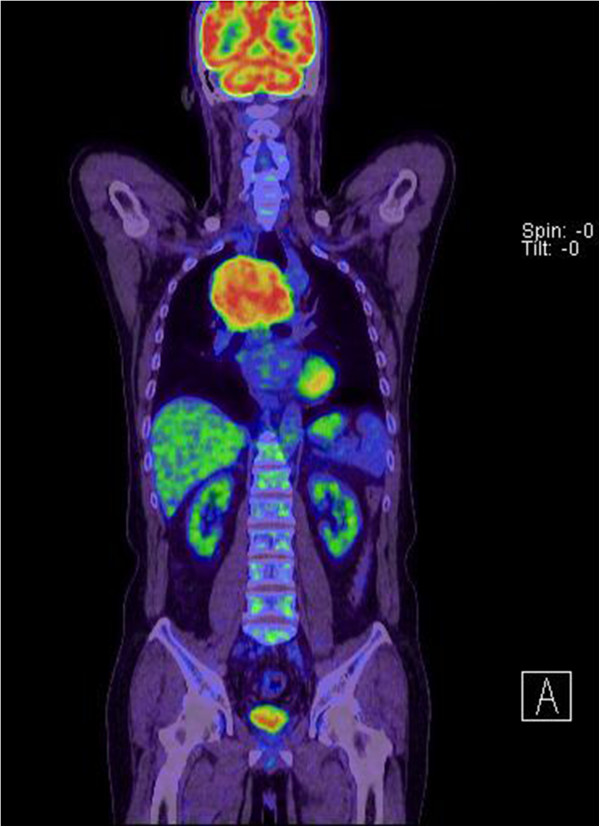
Hypermetabolic activity (SUVmax: 9.9) was shown by positron emission tomography.

A 63-year-old male was referred to our care for mediastinal widening by chest X-ray. On routine physical examination, no abnormalities were found and laboratory tests were unremarkable. Endoscopy revealed only superficial gastritis. Computed tomography (CT) of the chest disclosed a well-defined mass of 9.4 × 8.9 cm in the upper thoracic esophagus, with heterogeneous density after injection of contrast. No necrosis or hemorrhage was evident. This tumor was obstructive, causing proximal esophageal dilatation and tracheal compression (Figure 1), without signs of invasion. A hypermetabolic appearance (maximum standardized uptake value [SUVmax]: 9.9) was noted by positron emission tomography (PET) (Figure 2). Remarkably, the patient had no complaint of dysphagia or dyspnea. A percutaneous needle aspiration was interpreted as a benign spindle cell tumor. Under general anesthesia, a double lumen endotracheal tube was placed and there were no difficulties during anesthesia.

Right lateral thoracotomy was performed via the fourth intercostal space. Pleural effusion and adhesions were absent. The 10 × 10 cm mass protruded into the retrotracheal space, assuming a dumbbell shape. It was firm, well-encapsulated, and hypervascular. A 3-cm vertical esophageal myotomy was performed at the tumor level followed by tumor enucleation, but the surgical plane skirting a mass of this size was difficult to define. During extensive mucosal dissection, a mucosal tear (1 × 1 cm) was sustained, requiring primary repair. The muscle layer was approximated with non-absorbable sutures and regional lymph nodes were also removed. There was no malignancy including the mass and lymph nodes on the frozen section.

**Figure 3:**
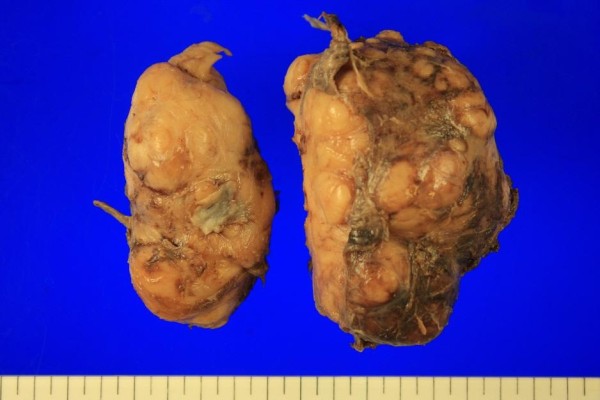
Macroscopic findings revealed multilobulated and dumbbell shape.

The resected tumor formed two distinct lobes measuring 9.5 × 7.0 × 6.5 cm and 8.8 × 5.0 × 5.5 cm, respectively (Figure 3); the overall weight was 243.2 g. Microscopically, a dense proliferation of spindle cells was seen, marked by a low mitotic count (1/10 high-power fields) and absence of necrosis. Sections of immunostained tumor were negative for smooth muscle actin, CD-117, desmin, and Ki-67, but S-100 protein was positive. All lymph nodes retrieved were clear. The patient recovered uneventfully, resuming a normal diet on postoperative day 7. During the 7-month follow-up, there was no evidence of recurrence or complications after operation.

#### Patient 2

**Figure 4:**
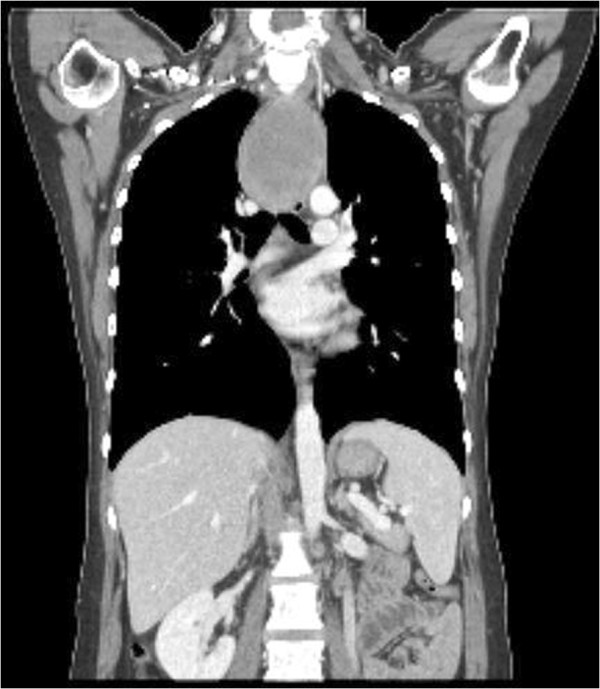
Coronal view of chest CT showed huge mass in the upper thoracic esophagus.

A 32-year-old female was admitted for mediastinal widening by chest X-ray. She had given birth 2 weeks previously and now complained of intermittent chest pain. Endoscopic ultrasonography and esophagography revealed a large, obstructive submucosal tumor of the upper thoracic esophagus (20–29 cm from incisor) without invasion. Endoscopic biopsy showed only inflammation, but a 6.0 × 8.5 × 4.0 cm homogenous mass of the upper thoracic esophagus was seen on chest CT, compressing the trachea (Figure 4). Again, a high metabolic tumor activity (SUVmax: 9.8) was evident by PET. The first clinical impression was of leiomyoma. A cervical approach was planned because the tumor was located between the thoracic inlet and carina. We experienced that enucleation was not easy through thoracotomy, as described earlier, and extensive mucosal dissection may lead to an increase in the mucosal injury through video-assisted thoracic surgery (VATS).

**Figure 5:**
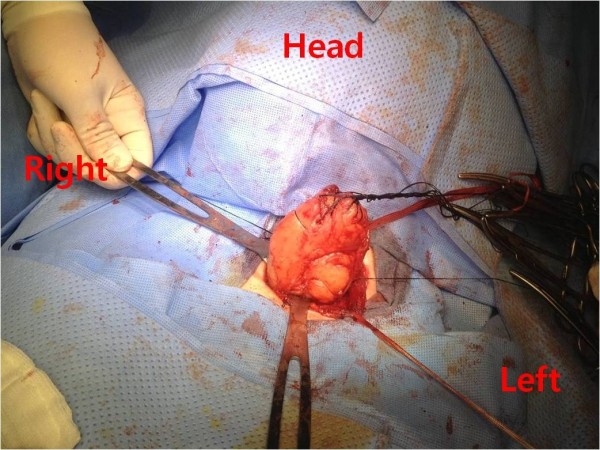
Enucleation of submucosal tumor by left cervical approach.

After general anesthesia, ventilation was difficult due to airway distortion. The tumor may have originated from the left esophageal wall, since radiolucency was visible at the right esophageal lumen. Submucosal tumor enucleation was attempted via the left cervical incision (Figure 5). We identified the mass by digital palpation. Myotomy was performed at the tumor level and the tumor was identified by its smooth surface. Several stay sutures were placed within the mass followed by blunt dissection. However, dense adherence at the site of prior endoscopic biopsy necessitated some in-continuity mucosal resection (1 × 1 cm) and repair. The mucosal perforation was easily identified. There was no malignancy on the frozen section. The muscle layer was closed with non-absorbable sutures.

**Figure 6:**
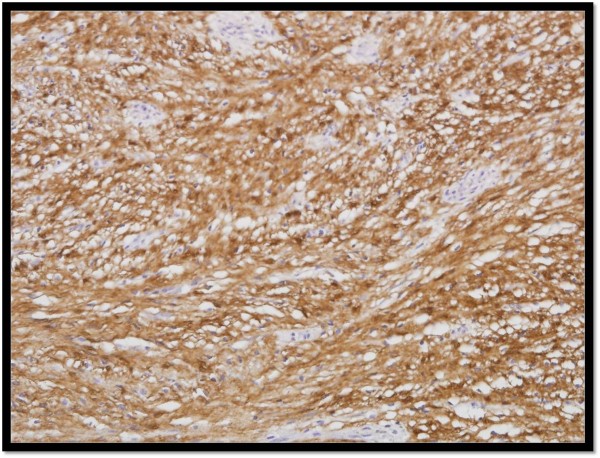
Palisading spindle cells, positive for S-100 protein, without mitosis and atypia (immunostaining).

The ovoid submucosal mass measured 8.7 × 5.9 × 2.4 cm. Its cut surface was yellowish and rubbery. Microscopically, an innocuous proliferation of spindle cells was observed, devoid of mitotic figures and positive for S-100 protein by immunostain (Figure 6). The patient did well following surgery and was discharged on postoperative day 10. During the 6-month follow-up, there was no evidence of recurrence or complications related to the operation.

### Comment

Schwannomas are the most common type of neurogenic tumor, derived from proliferating Schwann cells. Typically, the mediastinum is the site of origin. The majority of esophageal submucosal tumors are leiomyomas. Esophageal schwannomas are in fact exceedingly rare; approximately 30 cases have been reported in the literature [1]. The differential diagnosis is very difficult on the basis of imaging studies and endoscopic biopsies are sometimes misleading, given the mucosal barrier. Surgical indications include symptomatic lesions, lesions greater than 4 to 5 cm in diameter, suspicion of malignancy, and lesions with size progression. In the literature, esophageal schwannomas have a predilection for the upper thoracic segment [2]. The mean age of patients is 50 years, with a female predominance [3], and the most common symptom is dysphagia.

Schwannomas are usually benign, displaying one or two histologic patterns: Antoni A and B. Antoni A areas are compact zones with palisading of spindle cells, whereas loosely arranged tissue with variable cystic change and hemorrhage is designated Antoni B. Schwannomas tumors show positive immunostaining of S-100 protein and are negative for smooth muscle markers. Malignant transformation, albeit rare [4], is indicated by more frequent mitotic figures, necrosis, and cytologic irregularities. Although schwannomas may exhibit high metabolic activity by PET, implying malignancy, there is no real clinical correlation [5].

Tumor enucleation, as opposed to complete curative resection, is generally sufficient for benign schwannomas [6]. Due to sheer bulk, identifying a submucosal surgical plane can be challenging; but ensuring mucosal integrity is of utmost importance. In the literature, a predilection for upper thoracic esophagus is apparent, hence right thoracic access is often elected [1,2,4,5]. Nowadays, VATS has become more popular because it provides less pain and shorter recovery time than thoracotomy; however, a cervical approach is rare. The tumor location at the thoracic inlet is uncommon. Access to this lesion is not easy despite an open thoracotomy. In huge submucosal tumors, VATS may lead to an increase in mucosal injury during extensive mucosal dissection and overlooking of the injury site due to a huge mass. We achieved successful enucleation for large benign submucosal tumors via a cervical approach, creating a smaller incision and inflicting less pain than standard thoracotomy. On the other hand, the surgical view by this route was sometimes poor with large tumors. However, stay sutures were performed within the mass, mucosal dissection is possible during gentle retraction of the tumor. The cervical approach is an alternative modality. If malignancy is suspected, radical surgery (esophagectomy and lymph node dissection) is needed to prevent recurrence or nodal metastasis [4].

### Conclusions

Large esophageal schwannomas were successfully treated by enucleation. Despite the short-term encouraging outcomes, extended follow-up is mandatory due to the rarity of these neoplasms.

### Consent

Written informed consent was obtained from the patients for publication of this case report and images.

## Abbreviations

CT: Computed tomography; PET: Positron emission tomography; SUVmax: Maximum standardized uptake value; VATS: Video-assisted thoracic surgery.

## Competing interests

The authors declared that they have no competing interests.

## Authors’ contributions

HWJ: 1st. author, review of medical records, writing. KSK: review of medical record. KYH: revision. JKP: corresponding. All authors read and approved the final manuscript.
